# 初诊非APL急性髓系白血病合并DIC 1例报告及文献复习

**DOI:** 10.3760/cma.j.cn121090-20241129-00499

**Published:** 2024-12

**Authors:** 萌 周, 佳 陈, 悦 韩

**Affiliations:** 苏州大学附属第一医院，苏州 215006 The First Affiliated Hospital of Soochow University, Suzhou 215006, China

**Keywords:** 急性白血病, 弥散性血管内凝血, 非早幼粒细胞白血病, Acute leukemia, Disseminated intravascular coagulation, Non-acute promyelocytic leukemia

## Abstract

**目的:**

弥散性血管内凝血（DIC）常见于初诊的急性早幼粒细胞白血病（APL），死亡率极高，通过报道1例非APL急性髓系白血病并发DIC的成功救治，旨在提高临床医师对非APL中DIC的认识。

**方法:**

回顾1例AML（非APL）合并DIC患者的详细诊治过程并复习相关文献。

**结果:**

在早期识别DIC的基础上，通过适时合理的化疗和充足的支持治疗，该患者成功脱离DIC并达到疾病缓解，后续通过造血干细胞移植达到长期生存。

**结论:**

非APL合并DIC病情危重，需要早期识别和及时干预。

## Abstract (en)

**Objective:**

Disseminated intravascular coagulation（DIC）is common in newly diagnosed acute promyelocytic leukemia（APL）, with a high mortality rate. This case report focuses on a successful treatment of non-APL complicated with DIC, aiming to enhance clinical physicians' awareness and diagnostic skills in managing non-APL complicated by DIC.

**Methods:**

The diagnosis and treatment of a non-APL patient with DIC was detailed and related literature was reviewed.

**Results:**

With the help of early identification of DIC, chemotherapy and supportive treatment, the patient successfully recovered from DIC and achieved complete remission after induction therapy. He achieved long-term survival via subsequent hematopoietic stem cell transplantation.

**Conclusion:**

Non-APL patients with DIC are critically severe and early identification and timely intervention are necessary for them.

弥散性血管内凝血（DIC）常见于初诊的急性早幼粒细胞白血病（APL），死亡率极高，本文通过报道1例非APL急性髓系白血病（AML）并发DIC的成功救治，旨在提高临床医师对非APL中DIC的认识。

## 病例资料

患者，男，44岁，主因“发热伴咽喉疼痛半个月”于我院门诊就诊。患者既往有高血压病史数年，口服缬沙坦治疗，血压控制在110～130/70～90 mmHg，否认糖尿病、冠心病等慢性病史，否认乙肝、结核等传染病史，否认手术、外伤、输血史，否认食物药物过敏史。

2020年3月10日患者无明显诱因出现发热伴咽痛，就诊于当地医院，查血常规提示WBC 274.9×10^9^/L，HGB 111 g/L，PLT 32×10^9^/L。外周血涂片：原始细胞占97％，后就诊于上级医院，骨髓细胞形态和白血病免疫分型均提示AML，予以白细胞单采、羟基脲降低肿瘤负荷，血制品输注等支持治疗。现患者为求进一步诊治，于2020年3月24日入住我科。入院查体：中度贫血貌，全身多处瘀斑，双下肢2处皮下暗紫色结节，最大处直径约3 cm，质硬，有触痛，局部皮温升高，胸骨压痛（−），肝脾肋缘下未触及，余一般体格检查无明显异常。

入院后完善骨髓检查，形态：AML-M_2_可能，原始细胞占87％；白血病免疫分型：分析92.6％的幼稚细胞，为髓系抗原表达；PML/RARA-FISH：阴性；染色体：仅见5个分裂象，核型分析未见异常；多重PCR：未检测到白血病相关融合基因，WT1 10 422拷贝/10000 abl拷贝，EVI1<10拷贝/10000 abl 拷贝；二代测序：NPM1、FLT3-ITD阳性，故明确诊断为AML。患者入院血常规示：PLT 20×10^9^/L，WBC 198.15×10^9^/L，HGB 95 g/L，中性粒细胞计数5.75×10^9^/L；2020年3月24日查血凝七项组套（急）：部分凝血活酶时间35.2 s，D-二聚体>20 µg/ml，纤维蛋白降解物>150 mg/L，凝血酶原时间16.7 s，纤维蛋白原1.58 g/L，根据以上结果，患者中国弥散性血管内凝血诊断积分系统（CDSS）评分达到6分，ISTH DIC评分5分，均已达到DIC的诊断标准。入院后次日即行白细胞单采降低肿瘤负荷，术后血常规：WBC 77×10^9^/L，HGB 63 g/L，PLT 14×10^9^/L；3月26日患者白细胞再次反弹，且出现发热，热峰近40 °C，为保证患者安全，暂缓行白细胞单采，即刻予地西他滨联合IAG方案进行诱导治疗，同时予羟基脲进行降低白细胞治疗（具体流程见图1），辅以水化碱化尿液、成分血输注及抗感染等治疗措施。

**图1:**
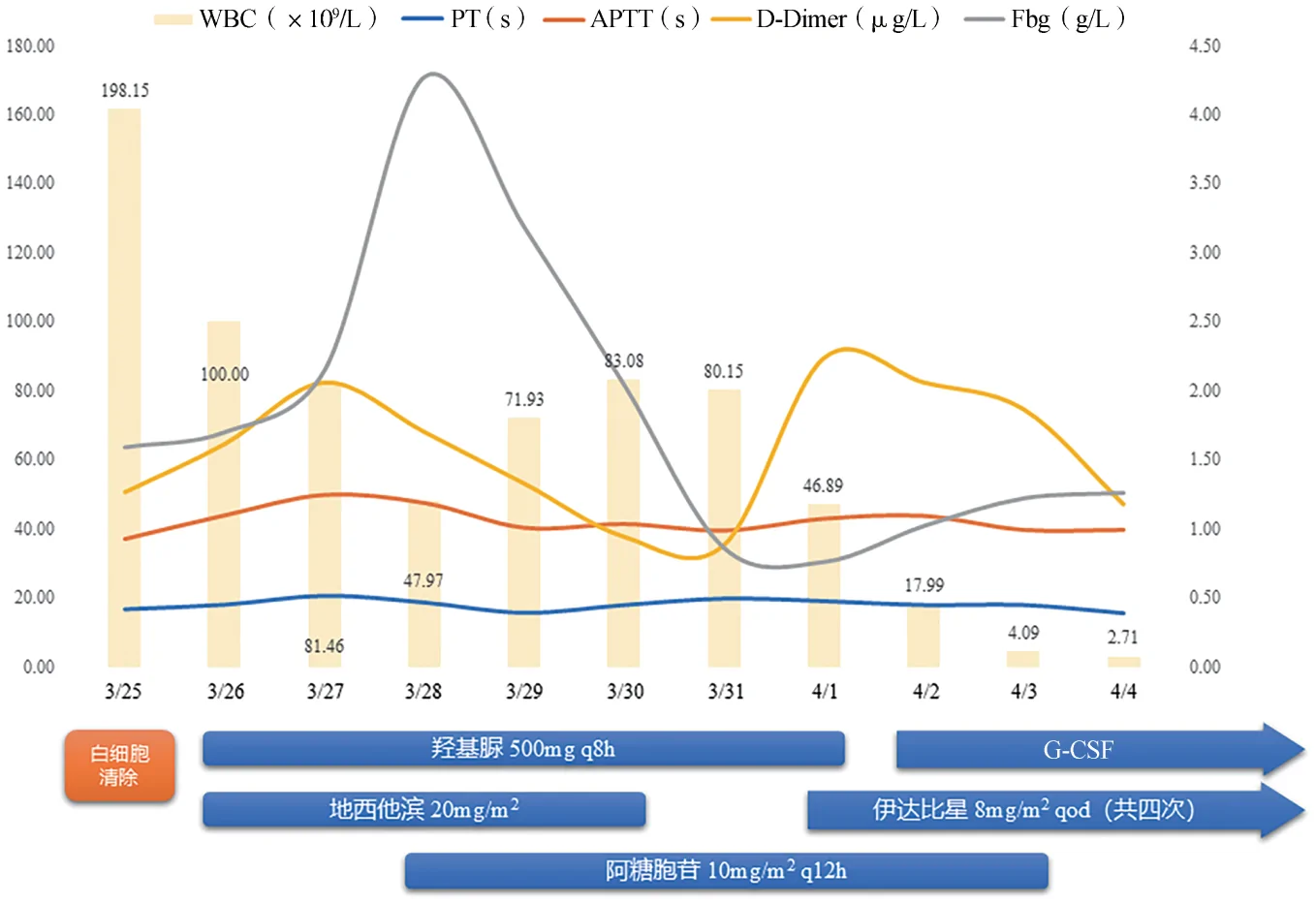
患者治疗过程及血凝的变化曲线

3月27日患者复测血常规：WBC 81.46×10^9^/L，HGB 54 g/L，PLT 6×10^9^/L；DIC（组套）：活化部分凝血活酶时间49.8 s，血浆凝血酶原时间20.5 s，纤维蛋白降解物223.67 µg/ml，D-二聚体82.45 µg/ml，此时CDSS评分达到8分，提示DIC仍然在进展，予升级抗生素控制感染、成分血输注、加强水化碱化控制肿瘤溶解综合征，同时予对症处理保护脏器（此时肌酐114.8 µmol/L）。3月28日患者白血病计数进一步下降，但是仍有高热，且出现嗜睡等，考虑与肿瘤溶解相关。血常规：WBC 47.97×10^9^/L，HGB 38 g/L，PLT 10×10^9^/L；血凝组套：纤维蛋白降解物>150 mg/L，凝血酶原时间18.6 s，部分凝血活酶时间47.5 s，CDSS评分依然为7分，治疗上无明显调整，着重血制品的输注和脏器功能的支持。3月29日患者神志转清，体温控制，CDSS评分为6分，凝血酶原时间与部分凝血活酶时间接近正常，但总体依然处于纤溶亢进的状态，考虑患者白细胞不断反跳，予加用小剂量阿糖胞苷皮下注射降低肿瘤负荷。3月30日和31日患者未再发热，且肾功能逐步恢复至正常。4月1日患者血凝再次出现恶化，表现为D-二聚体的急剧升高、凝血酶原时间的再度延长，以及纤维蛋白原的显著下降，且出现3P试验阳性，CDSS评分再次达到8分，考虑患者DIC再次加剧，合并出现了消耗性低凝状态。结合患者白细胞的变化情况，同日加用伊达比星进行诱导治疗，辅以血浆、冷沉淀、纤维蛋白原的输注，4月2日CDSS评分无明显下降，但D-二聚体、部分凝血活酶时间出现了不同程度的下降，纤维蛋白原有所回升；4月3日CDSS评分下降为7分，3P试验转阴；4月4日CDSS评分进一步下降为6分；4月8日伴随着D-二聚体的回落，患者CDSS评分低于6分，脱离DIC状态。后续该患者的治疗相对比较顺利，粒细胞缺乏期没有发热及出血的表现，诱导后评估本病缓解，经巩固治疗1个疗程后行子供父单倍体造血干细胞移植，目前仍然存活。

## 讨论及文献复习

本文报道了1例初诊AML（非APL）合并有严重出凝血紊乱患者的救治过程。治疗初期，患者出现了以纤溶亢进为主要表现的DIC症状，当合并感染及肿瘤溶解后，CDSS评分居高不下，DIC不断反复，经过AML的诱导以及强有力的支持治疗后，患者转危为安，也为其后续行造血干细胞移植创造了条件。

众所周知，在AML的各个类型中，APL更容易出现致死性的出血事件。APL凝血功能障碍的主要表现，包括低纤维蛋白原血症、纤维蛋白降解产物水平升高、凝血酶原和凝血活酶时间延长，而且上述异常会随着细胞毒性药物的应用逐步加剧，导致严重的出血性并发症[1]。其机制尚未完全明确，普遍观点认为该过程源于内皮损伤和血液高凝，血管内凝血激活导致血小板和凝血因子的消耗，小血管内纤维蛋白沉积，从而导致各器官的供血不足[2]。APL的凝血异常以纤溶亢进为主要表现，临床表现为D-二聚体和纤维蛋白（原）降解产物水平极度升高，纤溶酶原和α2-抗纤溶酶大量消耗，从而导致纤溶酶α2-抗纤溶酶复合物水平的升高[3]。本例中患者初诊时的血凝异常也是纤维蛋白降解产物和D-二聚体显著升高的纤溶亢进，并且随着诱导治疗的开始，其DIC的表现逐步加重。造成其二者表现类似的原因，可能是初诊时白细胞水平的异常升高。高白细胞血症导致组织因子与Ⅶ因子形成复合物，后者激活了Ⅺ和Ⅹ因子[4]；同时，异常白血病细胞释放肿瘤促凝物质，不依赖Ⅶ因子而促进Ⅹ因子的活化[5]；此外，白血病细胞释放的炎症因子如TNF-α、白细胞介素-1β，也会作用于内皮细胞导致激活释放组织因子和t-PA抑制剂纤溶酶原激活剂1型抑制剂（PAI-1）[6]。通过以上三个途径共同作用激活DIC。

除了APL以外，初诊AML中最容易合并DIC的类型为急性单核细胞白血病，国内一项基于初诊时CDSS评分的研究显示，初诊时CDSS评分每增加1分，60 d死亡率增加39％，而初诊时CDSS评分≥6分患者的60 d死亡风险是CDSS<6分的1.89倍，国外的数据也显示，初诊时合并DIC，是AML（非APL）预后不良的因素，因此早期识别和干预显得尤为重要[7]–[8]。本例患者的成功救治得益于全程的血凝指标监控以及血栓弹力图的动态监测，以及充分及时的血制品干预，使CDSS评分逐步下降至最终脱离DIC状态。

近年来，也有报道某些具有特殊细胞遗传学改变的AML（非APL）初诊时更容易合并DIC，如KMT2A重排、annexin A2（ANXA2）突变阳性以及t（8;16）[9]–[11]。本例中，我们也对该患者进行了常规AML基因突变组套之外的出凝血基因组套测序，发现该患者合并有MASTL和PRKACG基因的突变。上述两种基因突变并未被广泛研究，但均提示有可能导致血小板的异常：携带MASTL突变的小鼠可通过抑制有丝分裂过程中PP2A-B55复合物的异常活化和血小板存活而发生血小板减少症[12]；纯合子PRKACG突变导致血小板形成严重缺陷，最终导致巨血小板减少症[13]。虽然没有直接的证据，但是我们有理由怀疑，这两种基因的突变有可能参与了血小板功能的异常从而促进了DIC的发生和发展。

总体而言，本例中对患者的救治是成功的，为患者的后续造血干细胞移植治疗创造了条件，但是我们也反思，如果在保证充分水化碱化的基础上，更早地使用去甲氧柔红霉素（如WBC低于50×10^9^/L即开始应用）和100 mg/m^2^的阿糖胞苷能否缩短患者DIC的持续时间？这需要在未来临床工作中进一步观察和实践。

总之，AML（非APL）初诊时合并DIC是疾病预后不良的标志之一，需要早期识别，并进行积极干预，方能改善患者的预后。
